# Generating High-Resolution CT Slices from Two Image Series Using Deep-Learning-Based Resolution Enhancement Methods

**DOI:** 10.3390/diagnostics12112725

**Published:** 2022-11-08

**Authors:** Heng-Sheng Chao, Yu-Hong Wu, Linda Siana, Yuh-Min Chen

**Affiliations:** 1 Department of Chest Medicine, Taipei Veterans General Hospital, Taipei City 112, Taiwan; 2 Faculty of Medicine, School of Medicine, National Yang Ming Chiao Tung University, Taipei City 112, Taiwan; 3 Research and Development III, V5 Technologies Co., Ltd., Hsinchu 300, Taiwan

**Keywords:** super-resolution, deep learning, computed tomography, sagittal plane

## Abstract

Medical image super-resolution (SR) has mainly been developed for a single image in the literature. However, there is a growing demand for high-resolution, thin-slice medical images. We hypothesized that fusing the two planes of a computed tomography (CT) study and applying the SR model to the third plane could yield high-quality thin-slice SR images. From the same CT study, we collected axial planes of 1 mm and 5 mm in thickness and coronal planes of 5 mm in thickness. Four SR algorithms were then used for SR reconstruction. Quantitative measurements were performed for image quality testing. We also tested the effects of different regions of interest (ROIs). Based on quantitative comparisons, the image quality obtained when the SR models were applied to the sagittal plane was better than that when applying the models to the other planes. The results were statistically significant according to the Wilcoxon signed-rank test. The overall effect of the enhanced deep residual network (EDSR) model was superior to those of the other three resolution-enhancement methods. A maximal ROI containing minimal blank areas was the most appropriate for quantitative measurements. Fusing two series of thick-slice CT images and applying SR models to the third plane can yield high-resolution thin-slice CT images. EDSR provides superior SR performance across all ROI conditions.

## 1. Introduction

Medical imaging is an important activity in modern medical systems and the best means of diagnosing and treating patients [1]. For example, in the diagnosis and management of pulmonary diseases, medical imaging has evolved from simple planar film, and sonography, to computed tomography (CT) including low-dose CT. Chest CT is the best imaging modality for revealing pulmonary conditions such as emphysema, interstitial fibrosis, tumor masses and early lung cancer [2]. There is a growing demand for high-resolution (HR) thin-slice medical images for clinical applications to identify early lung cancer with sub-centimeter ground glass opacity, characterize lung fibrosis, determine the relationships between lung lesions and their surrounding structures, and provide quantitative measurements [3,4,5]. Thin-slice CT images also provide adequate information for generating navigation guides for bronchoscopy [6]. Based on the development of modern CT scanners, we achieved enhanced contrast, higher resolution, and reduced radiation doses.

Image resolution and slice thickness must inevitably be balanced with the file size of medical images, which impacts the cost of storage, speed of file exchange on the intranet or across institutions, and human resources required for interpretation. Although the original data from modern scanners can be reconstructed into thin-slice images, most images in the picture archiving and communication system (PACS) are not thin-slice images, meaning they have low spatial resolution in the Z plane. This system can reduce storage demand, facilitate file transfer, and reduce radiologist workload. However, for many clinical image-based tasks, physicians require not only fine spatial resolution along a particular plane, but also sufficient resolution in all directions to perform multi-planar reformatting, 3D imaging, or virtual volume rendering [5,6]. Therefore, there is a gap between special clinical image applications and image quality in the PACS.

Single-image super-resolution (SR) has been developed for decades in many industries [7]. SR was initially used in optical imaging, such as astronomy [8]. With a single image SR coupled with low-resolution (LR) numerical simulations, researchers can achieve SR simulation for real-time prediction in micrometeorology and the resolution can be improved by advanced numerical methods [9,10]. Current single-image SR based on deep learning methods can achieve acceptable performance and image quality on datasets containing natural images [11,12]. The method has also been applied to medical images to increase 2D resolution [13,14,15]. Its implementations include the SR convolutional neural network (SRCNN), the enhanced deep residual network (EDSR), the SR generative adversarial network (SRGAN) and many other modified SR algorithms for monochrome medical images. SR methods have also been applied to generate HR 3D medical images, particularly for magnetic resonance imaging (MRI) [16,17,18,19]. In the literature, the qualities of deep-learning-based SR-reconstructed HR 3D slices have been reported to be much higher than those of simple interpolations. However, existing 3D SR techniques use one series of CT or MRI images as the image source. Additionally, the LR images used in previous studies were simulated based on original HR images, which is not realistic. A clinical chest CT study contains several series of thick slices of different orthogonal planes. To obtain high-quality, thin-slice SR images, it is necessary to take advantage of all the information in a clinical image study.

In this study, we combined two thick CT images (5 mm thickness in both the axial and coronal series) into a single interpolated 3D LR image. Then, we applied different deep learning SR methods on the third orthogonal plane to generate thin-slice SR CT images. We hypothesized that after aligning the two planes, applying a deep learning SR technique on the third plane could obtain the best quality, regardless of the SR model. We also evaluated the impact of regions of interest (ROIs) on quantitative measurements and we discuss important points to be aware of in further medical image research. Our study provides a new concept to build SR CT images. The upscaled CT images can be used in clinical service and facilitate various types of longitudinal image-based research.

The remainder of this document is organized as follows. Section 2 describes the materials and methods. Section 3 presents the results of our experimental evaluations. Section 4 discusses the implications of the results presented in Section 3. Finally, Section 5 summarizes our conclusions.

## 2. Materials and Methods

Four SR models were applied in our study. In Section 2.1.1 to Section 2.1.4, we present a simple description of each network model and its modifications. Section 2.2 to Section 2.5 describe the Digital Imaging and Communications in Medicine (DICOM) data collection process, the process for generating LR DICOM data, and the training and testing procedure.

### 2.1. Description of Artificial Intelligence (AI) Network Models and their Modification

#### 2.1.1. SRCNN (Super-Resolution Convolutional Neural Network)

**Figure 1:**
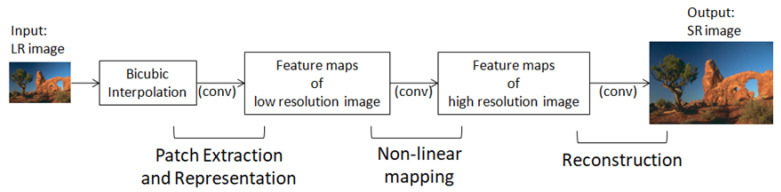
Flow for producing SR images using SRCNN.

SRCNN was the first CNN adapted to image SR reconstruction [11]. Its network is simple and consists of three components for patch extraction and representation, nonlinear mapping, and reconstruction. Figure 1 presents the SR flow in SRCNN. An input LR image is resized through bicubic interpolation to match the target size (i.e., SR size). Then, it is passed through the SRCNN to obtain an SR image. SRCNN is simple, fast, and provides good quality compared to classical computer vision algorithms for SR.

In this study, the resolution of our LR and HR images was the same, so we skipped the interpolation step and applied SRCNN directly.

#### 2.1.2. VDSR (Very Deep Super Resolution)

**Figure 2:**
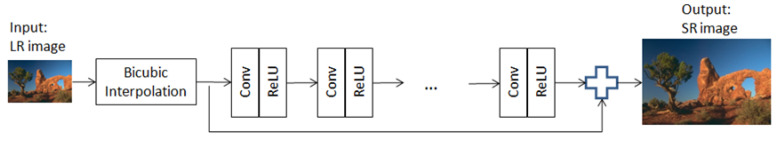
Flow for producing SR images using VDSR.

Kim et al. found that if a network has more convolution layers, it can provide better accuracy [20]. In the VDSR network, there are 20 layers of network depth. However, a deep-depth network typically encounters convergence problems during training. Therefore, the authors used residual learning and gradient clipping to overcome this issue. LR and HR images share significant low-frequency information, so training a model to learn the differences (i.e., high-frequency information) between LR and HR is advantageous. Figure 2 presents the flow for producing SR images using VDSR.

Similar to the SRCNN described above, because our LR and HR images have the same resolution, bicubic interpolation was omitted here.

#### 2.1.3. SRResNet (Generator Network for SRGAN)

**Figure 3:**
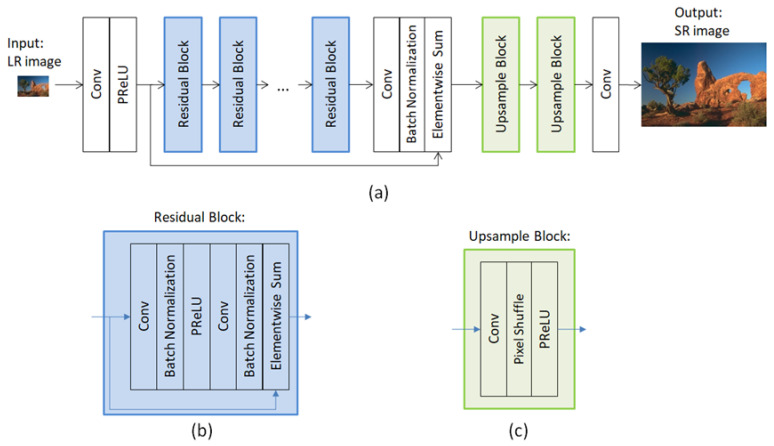
(**a**) Structure of the SRResNet network. (**b**) Residual block structure of SRResNet. (**c**) Upsampling block structure of SRResNet.

SRResNet is the generator for SRGAN [21], and we adopted this deep learning model as one of the evaluation networks. SRResNet is based on the concept of a residual network and contains 16 residual blocks. Figure 3 presents the SRResNet network. SRResNet applies pixel shuffling (subpixel convolution) for upsampling the feature map size.

In SRResNet, the upsampling block plays the role of changing the LR size to match the HR size. In this study, because our LR images had the same resolution as the HR images, we modified the upsampling block to provide 1:1 outputs, and removed the secondary upsampling block.

#### 2.1.4. EDSR (Enhanced Deep Residual Network)

**Figure 4:**
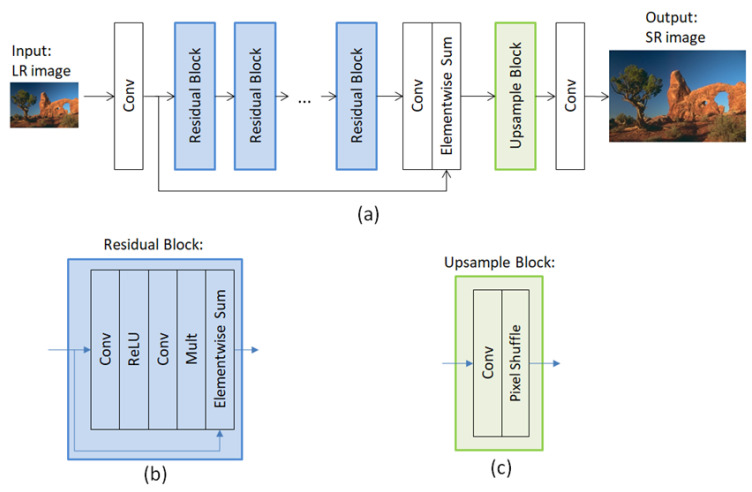
(**a**) Structure of EDSR. (**b**) Residual block structure of EDSR. (**c**) Upsample block structure of EDSR.

EDSR is based on SRResNet. Lim et al. performed some modifications on the original network and achieved enhanced results [22]. The EDSR structure is presented in Figure 4. Compared to SRResNet, EDSR removes the batch normalization layer because although batch normalization can normalize features in a SR task, preserving original features instead of normalizing them can yield enhanced detail in SR images. Additionally, Lim et al. found that when the number of feature maps increases, network training becomes more numerically unstable. Therefore, they applied a residual scaling factor of 0.1 to each residual block to overcome this issue.

In the original EDSR network, there are 16 residual blocks. In our study, we modified this from 16 to 32 residual blocks. Similar to SRResNet, we modified the upsampling block for 1:1 outputs because our LR and HR images had the same resolution.

### 2.2. Data Collection

Deidentified paired axial-plane images from chest CT scans and corresponding coronal-plane images from the same studies were collected. A portion of the CT images came from two retrospective studies, and the waving of informed consent was approved by the Institutional Review Board of Taipei Veterans General Hospital (TPEVGH IRB No.: 2019-07-046BC, 2021-04-014BC). The remaining CT images came from a study evaluating interstitial lung disease and their use was approved by the Institutional Review Board of the Taipei Veterans General Hospital where the participants provided informed consent (TPEVGH IRB No.: 2017-07-010CC). All CT studies were saved in the original DICOM format from the PACS. The deidentification process involved the elimination of all names and identifiers of patients in DICOM tags. All dates were modified to January 1st in the same year. We also generated new universal identifiers (UIDs) for each series. Beyond the CT images, we did not collect any other clinical data from the original studies. The CT scanner manufacturers corresponding to the images included Philips (Amsterdam, The Netherlands), Siemens Healthcare GmbH (Erlangen, Germany), and Toshiba (now Canon Medical Systems Corp. (Otawara, Japan)). The parameters of CT scanners of each case are listed in the Supplementary Table S1.

### 2.3. Image Preprocessing Process

For each CT scan, we reserved axial-plane slices of 1 mm and 5 mm in thickness, as well as a coronal-plane slice of 5 mm in thickness. If the axial-plane and coronal-plane were not right orthogonal to each other, then the CT set was discarded. Table 1 summarizes the requirements for collecting CT sets from DICOM.

**Figure 5:**
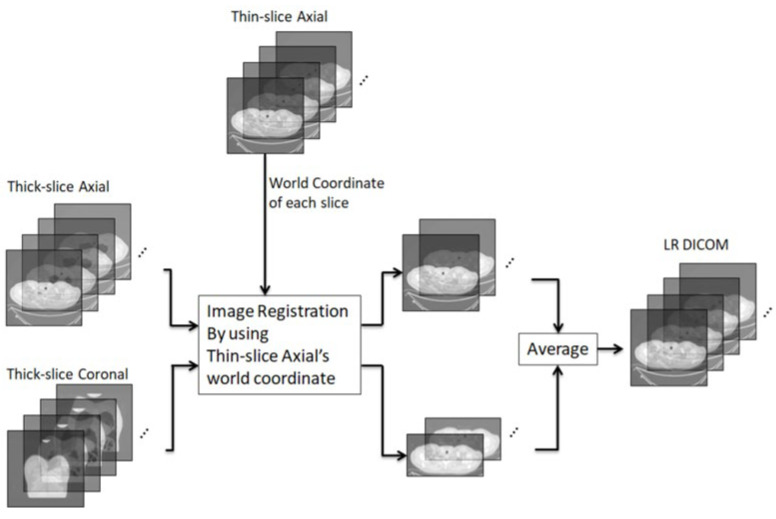
Process flow for generating LR DICOM data. From right to left of the figure, we performed image registration for the axial thick slices and coronal thick slices. Then, we combined the slices through averaging in 3D space to generate LR DICOM. The LR DICOM serves as the input for training and testing SR models.

In the image preprocessing process, our goal was to generate LR DICOM to serve an as input when training SR models. We used the world coordinates of axial thin slices to perform image registration in 3D space for the axial thick slices and coronal thick slices, and then combined the slices by averaging to generate LR DICOM data. LR DICOM data contains more details compares to axial thick slices. Figure 5 presents the process flow for generating LR DICOM data.

All 35 sets of lung CT DICOM data were processed in this manner to generate LR DICOM data. The thin-slice DICOM data in the axial plane were used as HR data. We split the 35 datasets into training, evaluation, and testing sets. Eleven datasets were used as training data and two datasets were used for evaluation during training. The remaining 22 datasets were reserved as testing data.

### 2.4. SR Model Selection and Training

**Figure 6:**
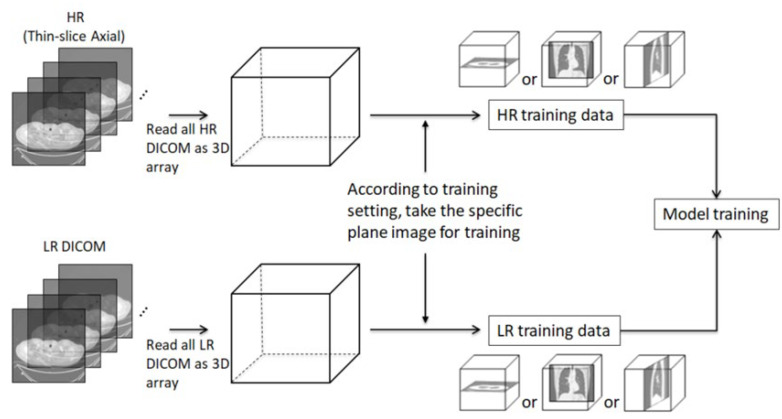
Diagram of model training for a specific plane.

In the training procedure, we read each pair of HR and LR DICOM data and stacked the images into a 3D array. According to the training settings, the pairs of HR and LR images were taken from a specific plane (i.e., axial, coronal, or sagittal) in the corresponding 3D array to act as inputs to train the model. Figure 6 presents a diagram of the training process.

After the HR and LR images were selected, we cropped random 96 × 96 pixel patches from the images. SRCNN, VDSR, SRResNet, and EDSR were trained using different parameters according to our training experience. The type of loss function was selected by referencing the original paper associated with each model. Table 2 summarizes the training settings for each model.

### 2.5. Testing Procedure

**Figure 7:**
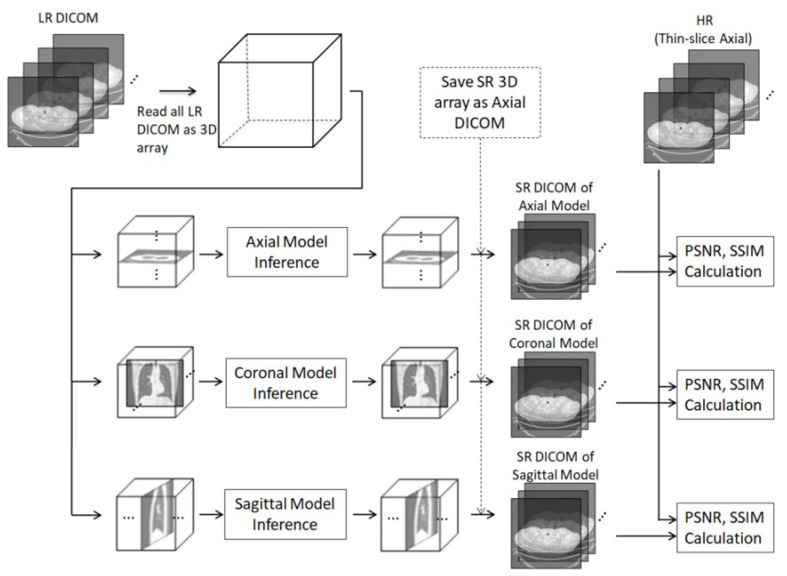
Testing PSNR and SSIM calculations.

We used 22 sets of HR and LR DICOM data to test each different model. Figure 7 presents a testing diagram with peak signal-to-noise ratio (PSNR) and structural similarity (SSIM) calculations.

### 2.6. Quantitative and Statistical Analysis

**Figure 8:**
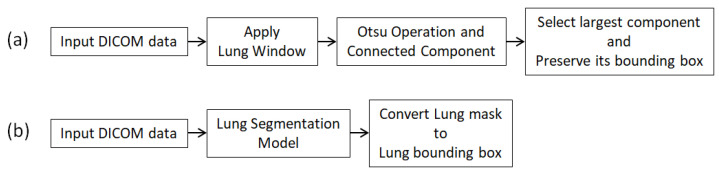
Generating desired ROI. (**a**) Body area; (**b**) Lung fields.

Two common metrics, namely PSNR and SSIM, were considered to compare quality between SR images generated using the deep learning models and ground-truth HR images. We also computed PSNR and SSIM values for segmented body and lung regions to focus on ROIs for physicians. The methods for ROI selection is illustrated in Figure 8 [23,24]. This type of evaluation can help reduce the effects of blank areas in medical images. The one-sided Wilcoxon signed-rank test was used to assess excellence scores between SR images (including LR images) generated by any two models. Boxplots were used to visualize PSNR and SSIM changes between model-generated SR images and ground-truth HR images. All numerical analysis and data preprocessing were performed using Python and R.

## 3. Results

We trained the deep learning models on a server with an AMD EPYC 7542 CPU and four NVIDIA A100 GPUs. The server was not a dedicated server and was shared by several colleagues. The number of GPUs for the training process was limited to two, and the training time could have had a greater variance. Table 3 lists the time required to train the different models. Table 4 lists the average processing times per SR DICOM slice inferred by different models and saved as DICOM files.

**Figure 9:**
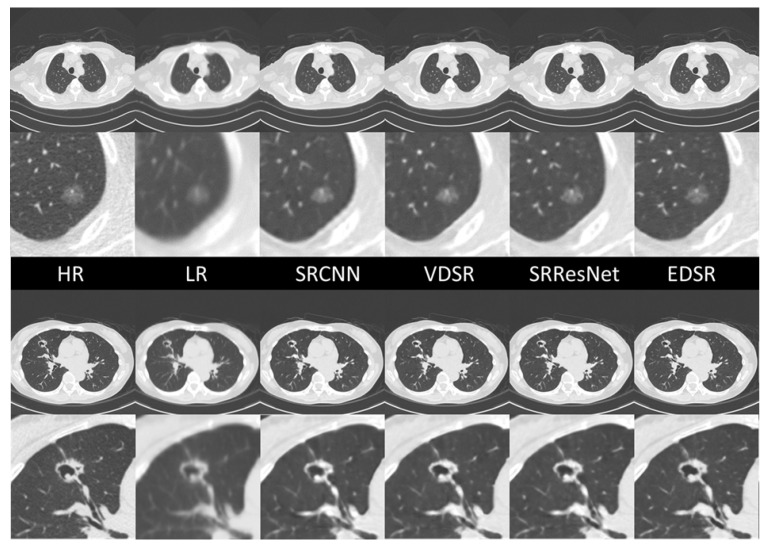
SR image samples of different AI models. All images were generated by applying SR models in the sagittal plan.

The ground-truth HR images, LR images, and sample comparisons for each SR DICOM slice generated along the sagittal axis are presented in Figure 9. The upper case is typical ground glass nodule that was confirmed to be early-stage lung cancer through surgical resection. All SR images maintain the ground glass features of this lesion, but only EDSR maintains the correct size and structure in terms of human visual quality. In the EDSR images, the fine structures of the surrounding lung markers are more clearly defined and correctly connected. The lower case is a cavitary lesion. Although the details of the upper border of the cavitary lesion are blurred in all SR images, the EDSR image preserves the arrangement of the proximal vessels and bronchi.

**Figure 10:**
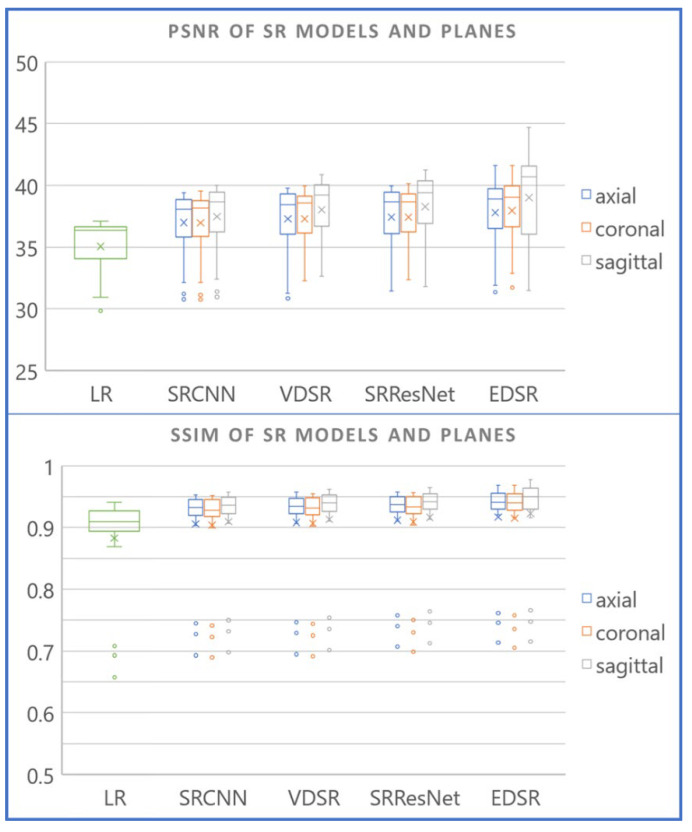
SSIM and PSNR of thin-slice (1 mm) SR CT images with different AI models and planes.

Table 5 lists the mean image quality metrics (including SSIM and PSNR) of different SR images compared to ground-truth HR images. Figure 10 presents a boxplot of the results for a visual comparison of the performance of each model. Model inferences for different planes are plotted near each other. LR results are also included in the graph for visual comparison. The SR models applied to the sagittal plane exhibited superior performance compared to those applies to the axial and coronal planes. Statistically, the PSNR and SSIM values of the SR images in the sagittal plane were significantly higher than those of another two planes, indicating that our hypothesis is correct. (see Table 6 and Table 7) EDSR was statistically better than other models, with the exception of SRResNet in PSNR.

**Figure 11:**
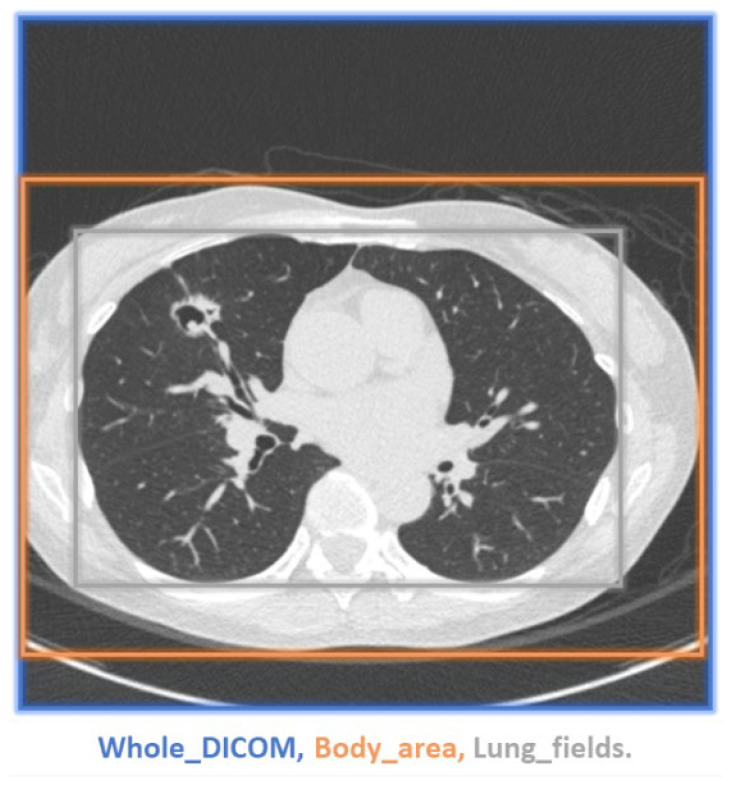
Demonstration of ROI selection. Blue box: whole DICOM image; Orange box: body area; Gray box: lung fields.

**Figure 12:**
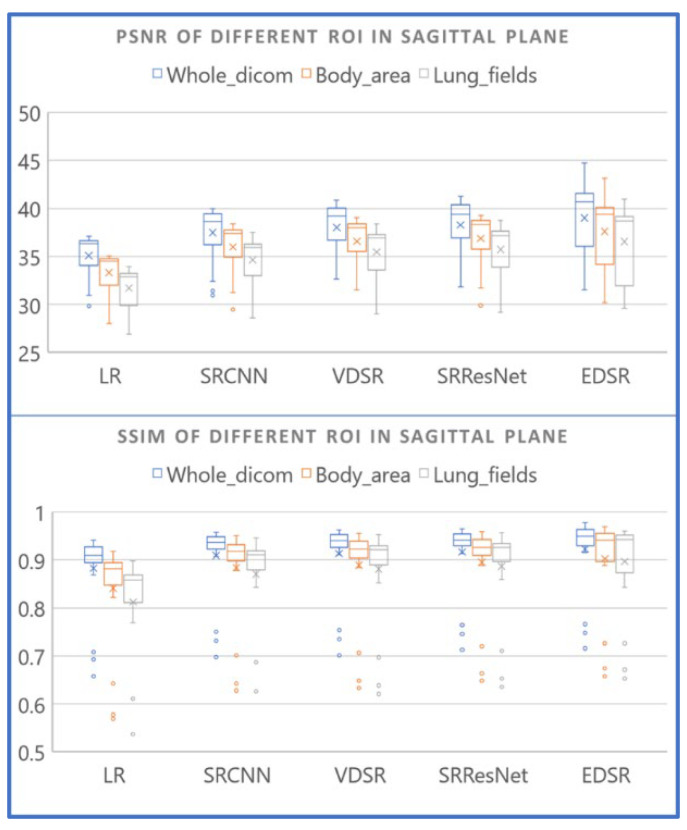
PSNR and SSIM values of ROIs for different AI models. All results represent applying the AI models to sagittal planes.

**Figure 13:**
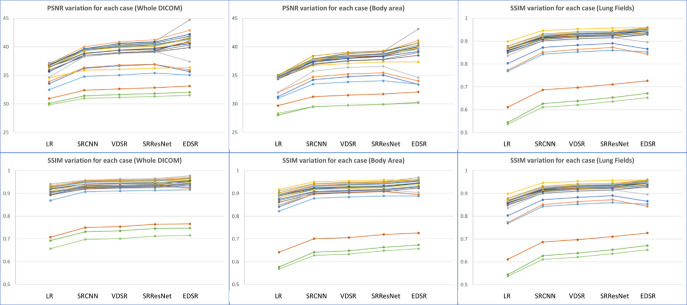
PSNR and SSIM variations for different ROIs and SR models. Each line represents the change in a single case. All results represent applying AI models to sagittal planes.

A demonstration of the proposed method for selecting an ROI is presented in Figure 11. While the ROI covered the entire images (blue box), we also selected bounding boxes for the body area (orange box) and lung fields (grey box). Smaller bounding boxes ignored most of the ambient air and focused on the structures we were interested in. As expected, more uniform black ambient air areas increase the PSNR and SSIM. When the entire image was included in the calculation, the PSNR and SSIM values were relatively high, whereas the lung field bounding boxes yielded the lowest values (see Table 7 and Figure 12). Regardless, applying EDSR to the sagittal plane yielded the best results for all three ROIs (see Table 8). Overall, the PSNR and SSIM increased in the same manner, regardless of ROI selection (see Figure 13). One can see that EDSR was superior to the other models on average, but sometimes, SRResNet performs better than EDSR.

## 4. Discussion

Deep learning techniques have been successfully applied to the field of medical imaging since 2014 [25]. In this study, we utilized SR methods to estimate HR medical image sequences from LR images. In contrast to most approaches in this area, we constructed a 3D LR data cube by combining two registered orthogonal image series (i.e., axial and coronal) from real-world clinical data. Furthermore, we compared the results of different SR models applied to different plane orientations of the 3D data cube. The PSNR and SSIM results of applying the SR models to the sagittal plane of the LR images were significantly better than the results of other orientations. The image quality achieved by the EDSR model was higher than that achieved by the other SR models. SRResNet also exhibited consistent quality gains across all test cases. The generated SR CT images may be used in other computer aided diagnosis tasks, such as pulmonary nodule detection and classification for early lung cancers [26,27,28]. Kazem, et al. found that high resolution SR images could assist the decision-makers in diagnosis [29].

When selecting ROIs in this study, we found that blank areas caused by ambient air can erroneously increase the scores of quantitative measures. Unlike natural images, medical images contain many blank areas. Some are attributed to the ambient air, and some are attributed to the scanning field of view. Ideally, the intensity differences in these regions are small and may increase similarity scores. The ROIs considered ranged from a 512 × 512 pixel DICOM image slice to a 32 × 32 window [30]. In our experiments, the higher the proportion of air and smaller the proportion of body tissue, the higher the PSNR and SSIM values. Additionally, regardless of which ROI was selected, SRResNet and EDSR achieved the best performance. Although the trend of change in the quality scores appeared constant regardless of ROI size, a general rule should be developed for selecting ROIs in future medical image SR studies.

Deep networks for SR are known to be influenced by the scale factor, which can be translated into the multiplier of a single voxel [17,19]. Small scale factors tend to yield better SR results. Our method combined two series of medical image planes to enrich the information in input LR images and reduce the theoretical scale factor. In this manner, the scale factor could be less than three on average, even when the original slice thickness was 5 mm.

Our study has several weaknesses. First, we only considered CT images in which the axial and coronal planes were right orthogonal to each other. Such images only accounted for one-tenth of our original data pool. CT images with arbitrary angles require 3-D registration and fusion. This process requires significant computational power. This condition requires further work and testing for integration into our SR workflow. Second, CT images may have different reconstruction kernels and we did not limit the reconstruction method of the input images. In most of the clinical cases collected, we merged 5 mm images with the so-called smooth kernels to predict the 1 mm axial images reconstructed by sharp kernels. Most previous studies used HR images to generate simulated LR images in which the CT reconstruction kernel or MR sequence remained the same [16,19,31]. There are also deep-learning-based image conversion methods for CT reconstruction kernels [32]. However, there are no articles discussing the influence of different reconstruction kernels for input images in SR problems. To observe the effects of input images with different combinations of reconstruction kernels, a much larger dataset is required before conclusions can be drawn. Finally, we did not have doctors to provide visual scores for output images. Many studies have derived human visual scores by various processes using different questions [30,33]. However, we performed some preliminary tests by ourselves. The SR images could be easily distinguished from HR and LR images, and senior physicians could point out differences between the images. The images wee not identical in all aspects, particularly in terms of the fine structures of artifacts. To derive more subjective visual scores, we require a thorough testing design, with cropping for ROIs, randomly selected images, and suitable questions.

On the basis of this study, several recommendations have been made to expand our current work in the future. Firstly, we can expand our collection of image files to balance the number of conventional CT, LDCT and contrast CT. Secondly, the SR algorithm proposed in the method should be updated for advanced performance. Thirdly, there are many new SR methods for medical images to enhance details between slices, so they need to be collected and compared to each other. Finally, since medical images are interpreted by physicians, it is necessary to apply a well-designed human visual score in further studies to meet clinical requirements.

## 5. Conclusions

We fused two orthogonal CT planes and applied several deep-learning-based SR methods to a third plane to generate HR slice CT images. Based on qualitative and quantitative comparisons, the EDSR model outperformed other deep-learning-based resolution enhancement methods. A reasonable ROI that contains a maximal ROI with minimal blank areas should be selected for reasonable quantitative measurements. Based on our real-world clinical dataset, the proposed method has excellent potential for clinical tasks and may also be beneficial for research tasks, such as updating old CT images to facilitate various types of longitudinal image-based research.

## Figures and Tables

**Table 1:** Requirements for each DICOM CT set used in our experiments.

Type	Plane	Slice Thickness (mm)	Spatial Interval between Neighbor Slice (mm)
Thin-slice	Axial	1~1.25	0.7~1.25 (and should ≤ Slice Thickness)
Thick-slice	Axial	5	5
Thick-slice	Coronal	5	5

**Table 2:** Training settings for each model.

Model	Patch Size	Batch Size	Batches Per Epoch	Total Epochs	Total Patches Trained	Initial Learning Rate	Loss
EDSR	96 × 96	16	7500	20	2,400,000	0.0001	L1
SRCNN	96 × 96	16	1000	200	3,200,000	0.0001	MSE
VDSR	96 × 96	16	1000	240	3,840,000	0.00001	MSE
SRResNet	96 × 96	16	1000	180	2,880,000	0.0001	MSE

**Table 3:** Training time.

Model	Number of GPUs	Number of Data Loader Threads	Training Time (Hours)
EDSR-Axial	2	16	34.09
EDSR-Coronal	2	16	34.02
EDSR-Sagittal	2	16	35.89
SRCNN-Axial	2	16	16.33
SRCNN-Coronal	2	16	13.00
SRCNN-Sagittal	2	16	13.11
VDSR-Axial	2	16	19.67
VDSR-Coronal	2	16	17.25
VDSR-Sagittal	2	16	16.55
SRResNet-Axial	2	16	16.53
SRResNet-Coronal	2	16	17.86
SRResNet-Sagittal	2	16	13.27

**Table 4:** Time required to generate each SR DICOM slice.

Model	Number of GPU	Processing Time (sec/slice) (Inference and Saving Results as DICOM Files)
EDSR-Axial	1	0.7415
EDSR-Coronal	1	0.5097
EDSR-Sagittal	1	0.5110
SRCNN-Axial	1	0.0836
SRCNN-Coronal	1	0.0704
SRCNN-Sagittal	1	0.0732
VDSR-Axial	1	0.1467
VDSR-Coronal	1	0.1271
VDSR-Sagittal	1	0.1398
SRResNet-Axial	1	0.1814
SRResNet-Coronal	1	0.1300
SRResNet-Sagittal	1	0.1273

**Table 5:** Mean image quality metrics of different SR images compared to the ground-truth HR images. Although LR is not an SR model, we duplicated the LR results in all three planes. (**a**) Mean PSNR of different SR models applied to the three planes. (**b**) Mean SSIM of different SR models applied to the three planes.

(a)					
	LR	SRCNN	VDSR	SRResNet	EDSR
axial	35.066	36.988	37.280	37.430	37.786
coronal	35.066	36.958	37.279	37.426	37.948
sagittal	35.066	37.479	38.026	38.281	39.021
**(b)**					
	**LR**	**SRCNN**	**VDSR**	**SRResNet**	**EDSR**
axial	0.8827	0.9058	0.9080	0.9118	0.9171
coronal	0.8827	0.9038	0.9067	0.9091	0.9149
sagittal	0.8827	0.9094	0.9132	0.9165	0.9227

**Table 6:** *p*-values for comparing PSNR (upper table) and SSIM (lower table) values between different SR models applied to different planes. The SR methods in rows are better than those in columns when the corresponding *p*-values after Bonferroni correction are less than 6.41 × 10^−4^ in a one-sided Wilcoxon signed rank test.

PSNR		LR	SRCNN	SRCNN	SRCNN	VDSR	VDSR	VDSR	SRResNet	SRResNet	SRResNet	EDSR	EDSR	EDSR
			Axial	Coronal	Sagittal	Axial	Coronal	Sagittal	Axial	Coronal	Sagittal	Axial	Coronal	Sagittal
LR			2.38 × 10^−7^	2.38 × 10^−7^	2.38 × 10^−7^	2.38 × 10^−7^	2.38 × 10^−7^	2.38 × 10^−7^	2.38 × 10^−7^	2.38 × 10^−7^	2.38 × 10^−7^	2.38 × 10^−7^	2.38 × 10^−7^	2.38 × 10^−7^
SRCNN	axial			1.38 × 10^−1^	2.38 × 10^−7^	4.77 × 10^−7^	2.14 × 10^−4^	2.38 × 10^−7^	2.38 × 10^−7^	1.07 × 10^−4^	2.38 × 10^−7^	4.71 × 10^−4^	2.38 × 10^−7^	2.38 × 10^−6^
SRCNN	coronal				2.38 × 10^−7^	6.03 × 10^−5^	2.38 × 10^−7^	2.38 × 10^−7^	4.77 × 10^−7^	2.38 × 10^−7^	2.38 × 10^−7^	2.96 × 10^−4^	2.38 × 10^−7^	1.19 × 10^−6^
SRCNN	sagittal					1.00	1.00	2.38 × 10^−7^	9.54 × 10^−1^	2.51 × 10^−1^	2.38 × 10^−7^	2.50 × 10^−2^	7.87 × 10^−6^	3.27 × 10^−5^
VDSR	axial						7.80 × 10^−2^	2.38 × 10^−7^	2.38 × 10^−7^	5.16 × 10^−3^	2.38 × 10^−7^	2.96 × 10^−3^	1.67 × 10^−5^	1.67 × 10^−5^
VDSR	coronal							2.38 × 10^−7^	2.94 × 10^−1^	2.38 × 10^−7^	2.38 × 10^−7^	1.04 × 10^−2^	2.38 × 10^−7^	1.67 × 10^−6^
VDSR	sagittal								1.00	1.00	2.38 × 10^−7^	8.40 × 10^−1^	9.17 × 10^−1^	4.71 × 10^−4^
SRResNet	axial									4.27 × 10^−2^	2.38 × 10^−7^	6.36 × 10^−3^	1.07 × 10^−4^	2.10 × 10^−5^
SRResNet	coronal										2.38 × 10^−7^	3.42 × 10^−2^	2.38 × 10^−7^	7.87 × 10^−6^
SRResNet	sagittal											9.95 × 10^−1^	1.00	5.16 × 10^−3^
EDSR	axial												1.94 × 10^−1^	1.62 × 10^−3^
EDSR	coronal													2.96 × 10^−4^
EDSR	sagittal													
**SSIM**		**LR**	**SRCNN**	**SRCNN**	**SRCNN**	**VDSR**	**VDSR**	**VDSR**	**SRResNet**	**SRResNet**	**SRResNet**	**EDSR**	**EDSR**	**EDSR**
			**Axial**	**Coronal**	**Sagittal**	**Axial**	**Coronal**	**Sagittal**	**Axial**	**Coronal**	**Sagittal**	**Axial**	**Coronal**	**Sagittal**
LR			2.38 × 10^−7^	2.38 × 10^−7^	2.38 × 10^−7^	2.38 × 10^−7^	2.38 × 10^−7^	2.38 × 10^−7^	2.38 × 10^−7^	2.38 × 10^−7^	2.38 × 10^−7^	2.38 × 10^−7^	2.38 × 10^−7^	2.38 × 10^−7^
SRCNN	axial			1.00	2.38 × 10^−7^	2.38 × 10^−7^	1.26 × 10^−2^	2.38 × 10^−7^	2.38 × 10^−7^	3.27 × 10^−5^	2.38 × 10^−7^	2.38 × 10^−7^	2.38 × 10^−7^	2.38 × 10^−7^
SRCNN	coronal				2.38 × 10^−7^	2.38 × 10^−7^	2.38 × 10^−7^	2.38 × 10^−7^	2.38 × 10^−7^	2.38 × 10^−7^	2.38 × 10^−7^	2.38 × 10^−7^	2.38 × 10^−7^	2.38 × 10^−7^
SRCNN	sagittal					1.00	1.00	2.38 × 10^−7^	2.10 × 10^−5^	5.25 × 10^−1^	2.38 × 10^−7^	4.53 × 10^−6^	2.38 × 10^−7^	4.77 × 10^−7^
VDSR	axial						9.44 × 10^−1^	2.38 × 10^−7^	2.38 × 10^−7^	5.74 × 10^−3^	2.38 × 10^−7^	1.19 × 10^−6^	4.77 × 10^−7^	4.77 × 10^−7^
VDSR	coronal							2.38 × 10^−7^	2.38 × 10^−7^	2.38 × 10^−7^	2.38 × 10^−7^	7.15 × 10^−7^	2.38 × 10^−7^	2.38 × 10^−7^
VDSR	sagittal								9.86 × 10^−1^	1.00	2.38 × 10^−7^	1.25 × 10^−3^	5.46 × 10^−4^	3.34 × 10^−6^
SRResNet	axial									1.00	2.38 × 10^−7^	1.67 × 10^−5^	8.36 × 10^−4^	3.34 × 10^−6^
SRResNet	coronal										2.38 × 10^−7^	3.34 × 10^−6^	2.38 × 10^−7^	2.38 × 10^−7^
SRResNet	sagittal											2.03 × 10^−1^	9.87 × 10^−1^	1.07 × 10^−4^
EDSR	axial												9.75 × 10^−1^	8.36 × 10^−4^
EDSR	coronal													1.03 × 10^−5^
EDSR	sagittal													

**Table 7:** Mean image quality metrics of different ROIs in SR images compared to the ground-truth HR images. (**a**) Mean PSNR of different ROIs in SR models. (**b**) Mean SSIM of different ROIs in SR models.

(a)					
	LR	SRCNN	VDSR	SRResNet	EDSR
whole-DICOM	35.066	37.479	38.026	38.281	39.021
body-area	33.314	35.987	36.590	36.865	37.609
lung-fields	31.709	34.640	35.459	35.730	36.558
**(b)**					
	**LR**	**SRCNN**	**VDSR**	**SRResNet**	**EDSR**
whole-DICOM	0.8827	0.9094	0.9132	0.9165	0.9227
body-area	0.8409	0.8838	0.8894	0.8950	0.9029
lung-fields	0.8124	0.8707	0.8811	0.8871	0.8966

**Table 8:** *p*-values for comparing PSNR and SSIM values between different SR models applied to different ROIs. The SR methods in rows are better than those in columns when the corresponding *p*-values after Bonferroni correction are less than 1.11 × 10^−3^ in a one-sided Wilcoxon signed rank test. We did not count the p-values for different methods applied to different ROIs because they have no practical meaning.

PSNR	LR	LR	LR	SRCNN	SRCNN	SRCNN	VDSR	VDSR	VDSR	SRResNet	SRResNet	SRResNet	EDSR	EDSR	EDSR
	Lung	Body	Whole	Lung	Body	Whole	Lung	Body	Whole	Lung	Body	Whole	Lung	Body	Whole
LR Lung_fields		2.38 × 10^−7^	2.38 × 10^−7^	2.38 × 10^−7^			2.38 × 10^−7^			2.38 × 10^−7^			2.38 × 10^−7^		
LR Body_area			2.38 × 10^−7^		2.38 × 10^−7^			2.38 × 10^−7^			2.38 × 10^−7^			2.38 × 10^−7^	
LR Whole_dicom						2.38 × 10^−7^			2.38 × 10^−7^			2.38 × 10^−7^			2.38 × 10^−7^
SRCNN Lung_fields					7.15 × 10^−7^	4.77 × 10^−7^	2.38 × 10^−7^			2.38 × 10^−7^			4.03 × 10^−5^		
SRCNN Body_area						4.77 × 10^−7^		2.38 × 10^−7^			2.38 × 10^−7^			7.32 × 10^−5^	
SRCNN Whole_dicom									2.38 × 10^−7^			2.38 × 10^−7^			3.27 × 10^−5^
VDSR Lung_fields								3.27 × 10^−5^	3.34 × 10^−6^	2.38 × 10^−7^			2.96 × 10^−3^		
VDSR Body_area									1.19 × 10^−6^		2.38 × 10^−7^			1.10 × 10^−3^	
VDSR Whole_dicom												2.38 × 10^−7^			4.71 × 10^−4^
SRResNet Lung_fields											3.27 × 10^−5^	1.67 × 10^−5^	1.64 × 10^−2^		
SRResNet Body_area												1.67 × 10^−6^		1.51 × 10^−2^	
SRResNet Whole_dicom															5.16 × 10^−3^
EDSR Lung_fields														4.94 × 10^−5^	7.87 × 10^−6^
EDSR Body_area															1.19 × 10^−6^
EDSR Whole_dicom															
**SSIM**	**LR**	**LR**	**LR**	**SRCNN**	**SRCNN**	**SRCNN**	**VDSR**	**VDSR**	**VDSR**	**SRResNet**	**SRResNet**	**SRResNet**	**EDSR**	**EDSR**	**EDSR**
	**Lung**	**Body**	**Whole**	**Lung**	**Body**	**Whole**	**Lung**	**Body**	**Whole**	**Lung**	**Body**	**Whole**	**Lung**	**Body**	**Whole**
LR Lung_fields		2.38 × 10^−7^	2.38 × 10^−7^	2.38 × 10^−7^			2.38 × 10^−7^			2.3 × 10^−7^			2.38 × 10^−7^		
LR Body_area			2.38 × 10^−7^		2.38 × 10^−7^			2.38 × 10^−7^			2.38 × 10^−7^			2.38 × 10^−7^	
LR Whole_dicom						2.38 × 10^−7^			2.38 × 10^−7^			2.38 × 10^−7^			2.38 × 10^−7^
SRCNN Lung_fields					4.77 × 10^−7^	2.38 × 10^−7^	2.38 × 10^−7^			2.38 × 10^−7^			3.34 × 10^−6^		
SRCNN Body_area						4.77 × 10^−7^		2.38 × 10^−7^			2.38 × 10^−7^			3.34 × 10^−6^	
SRCNN Whole_dicom									2.38 × 10^−7^			2.38 × 10^−7^			4.77 × 10^−7^
VDSR Lung_fields								1.81 × 10^−4^	4.77 × 10^−7^	2.38 × 10^−7^			1.28 × 10^−4^		
VDSR Body_area									4.77 × 10^−7^		2.38 × 10^−7^			1.67 × 10^−5^	
VDSR Whole_dicom												2.38 × 10^−7^			3.34 × 10^−6^
SRResNet Lung_fields											2.96 × 10^−4^	4.77 × 10^−7^	1.79 × 10^−2^		
SRResNet Body_area												4.77 × 10^−7^		4.64 × 10^−3^	
SRResNet Whole_dicom															1.07 × 10^−4^
EDSR Lung_fields														2.71 × 10^−2^	4.77 × 10^−7^
EDSR Body_area															4.77 × 10^−7^
EDSR Whole_dicom															

## Data Availability

Not applicable.

## Supplementary Materials

The following supporting information can be downloaded at: https://www.mdpi.com/article/10.3390/diagnostics12112725/s1, Table S1: Parameters of CT scanners of each case.

## References

[1] Fryback D.G., Thornbury J.R. (1991). The efficacy of diagnostic imaging. Med. Decis. Mak..

[2] Adeniji-Sofoluwe A.-T., Adekanmi A.-J., Efidi R. (2017). Imaging Findings in Chest Computed Tomography: Initial Experience in a Developing Country. Open J. Clin. Diag..

[3] Best A.-C., Meng J., Lynch A.-M., Bozic C.-M., Miller D., Grunwald G.-K., Lynch D.-A. (2008). Idiopathic pulmonary fibrosis: Physiologic tests, quantitative CT indexes, and CT visual scores as predictors of mortality. Radiology.

[4] Oh J.-Y., Kwon S.-Y., Yoon H.-I., Lee S.-M., Yim J.-J., Lee J.-H., Yoo C.-G., Kim Y.-W., Han S.-K., Shim Y.-S. (2007). Clinical significance of a solitary ground-glass opacity (GGO) lesion of the lung detected by chest CT. Lung Cancer.

[5] Achenbach T., Weinheimer O., Buschsieweke C., Heussel C.-P., Thelen M., Kauczor H.U. (2004). Fully automatic detection and quantification of emphysema on thin section MD-CT of the chest by a new and dedicated software. Fortschr. Geb. Röntgenstrahlen Nukl. Med..

[6] Matsumoto Y., Izumo T., Sasada S., Tsuchida T., Ohe Y. (2017). Diagnostic utility of endobronchial ultrasound with a guide sheath under the computed tomography workstation (ziostation) for small peripheral pulmonary lesions. Clin. Respir. J..

[7] Amanatiadis A., Andreadis I. (2009). A survey on evaluation methods for image interpolation. Meas. Sci. Technol..

[8] Puschmann K.G., Kneer F. (2005). On super-resolution in astronomical imaging. Astron. Astrophys..

[9] Onishi R., Sugiyama D., Matsuda K. (2019). Super-resolution simulation for real-time prediction of urban micrometeorology. Sola.

[10] Mahdy A.M.S. (2022). A numerical method for solving the nonlinear equations of Emden-Fowler models. J. Ocean Eng. Sci..

[11] Dong C., Loy C.-C., He K., Tang X. (2015). Image super-resolution using deep convolutional networks. IEEE Trans. Patt. Anal. Mach. Intell..

[12] Dong C., Loy C.-C., He K., Tang X. Learning a deep convolutional network for image super-resolution. Proceedings of the European Conference on Computer Vision.

[13] Pham C.-H., Ducournau A., Fablet R., Rousseau F. Brain MRI super-resolution using deep 3D convolutional networks. Proceedings of the IEEE Conference on International Symposium on Biomedical Imaging.

[14] Wei S., Wu W., Jeon G., Ahmad A., Yang X. (2020). Improving resolution of medical images with deep dense convolutional neural network. Concurr. Comput..

[15] Qiu D., Cheng Y., Wang X., Zhang X. (2021). Multi-window back-projection residual networks for reconstructing COVID-19 CT super-resolution images. Comput. Methods Programs Biomed..

[16] Zhang K., Hu H., Philbrick K., Conte G.-M., Sobek J.-D., Rouzrokh P., Erickson B.-J. (2022). SOUP-GAN: Super-resolution MRI using generative adversarial networks. Tomography.

[17] Du J., He Z., Wang L., Gholipour A., Zhou Z., Chen D., Jia Y. (2020). Super-resolution reconstruction of single anisotropic 3D MR images using residual convolutional neural network. Neurocomputing.

[18] Sánchez I., Vilaplana V. (2018). Brain MRI super-resolution using 3D generative adversarial networks. arXiv.

[19] Zhao C., Carass A., Dewey B.-E., Prince J.-L. Self super-resolution for magnetic resonance images using deep networks. Proceedings of the IEEE Conference on International Symposium on Biomedical Imaging.

[20] Kim J., Lee J.-K., Lee K.-M. Accurate image super-resolution using very deep convolutional networks. Proceedings of the IEEE Conference on Computer Vision and Pattern Recognition.

[21] Ledig C., Theis L., Huszár F., Caballero J., Cunningham A., Acosta A., Aitken A., Tejani A., Totz J., Wang Z. Photo-realistic single image super-resolution using a generative adversarial network. Proceedings of the IEEE Conference on Computer Vision and Pattern Recognition.

[22] Lim B., Son S., Kim H., Nah S., Mu Lee K. Enhanced deep residual networks for single image super-resolution. Proceedings of the IEEE Conference on Computer Vision and Pattern Recognition.

[23] van Rikxoort E.-M., de Hoop B., Viergever M.-A., Prokop M., van Ginneken B. (2009). Automatic lung segmentation from thoracic computed tomography scans using a hybrid approach with error detection. Med. Phys..

[24] Hofmanninger J., Prayer F., Pan J., Röhrich S., Prosch H., Langs G. (2020). Automatic lung segmentation in routine imaging is primarily a data diversity problem, not a methodology problem. Eur. Radiol. Exp..

[25] Kingma D.-P., Ba J. (2014). Adam: A method for stochastic optimization. arXiv.

[26] Nishio M., Sugiyama O., Yakami M., Ueno S., Kubo T., Kuroda T., Togashi K. (2018). Computer-aided diagnosis of lung nodule classification between benign nodule, primary lung cancer, and metastatic lung cancer at different image size using deep convolutional neural network with transfer learning. PLoS ONE.

[27] Liu X., Hou F., Qin H., Hao A. (2018). Multi-view multi-scale CNNs for lung nodule type classification from CT images. Pattern Recognit..

[28] Setio A.-A.-A., Ciompi F., Litjens G., Gerke P., Jacobs C., Van Riel S.-J., Wille M.-M.-W., Naqibullah M., Sánchez C.-I., Van Ginneken B. (2016). Pulmonary nodule detection in CT images: False positive reduction using multi-view convolutional networks. IEEE Trans. Med. Imaging.

[29] Kazem R.-A.H., Suad J., Abdulbaqi A. (2021). Super-resolution using 3d convolutional neural networks in CT scan image of COVID19. Turk. J. Comput. Math. Educ..

[30] Chaudhari A.-S., Fang Z., Kogan F., Wood J., Stevens K.-J., Gibbons E.-K., Lee J.-H., Gold G.-E., Hargreaves B.-A. (2018). Super-resolution musculoskeletal MRI using deep learning. Magn. Reason. Med..

[31] Zhao C., Dewey B.-E., Pham D.-L., Calabresi P.-A., Reich D.-S., Prince J.-L. (2020). SMORE: A self-supervised anti-aliasing and super-resolution algorithm for MRI using deep learning. IEEE Trans. Med. Imaging.

[32] Choe J., Lee S.-M., Do K.-H., Lee G., Lee J.-G., Lee S.-M., Seo J.-B. (2019). Deep learning–based image conversion of CT reconstruction kernels improves radiomics reproducibility for pulmonary nodules or masses. Radiology.

[33] Arnold T.-C., Baldassano S.-N., Litt B., Stein J.-M. (2022). Simulated diagnostic performance of low-field MRI: Harnessing open-access datasets to evaluate novel devices. Magn. Reason. Imaging.

