# Supplementary material for: Generating High-Resolution CT Slices from Two Image Series Using Deep-Learning-Based Resolution Enhancement Methods
Source: Diagnostics (Basel). 2022 Nov 8;12(11):2725. doi: 10.3390/diagnostics12112725 (PMC9689374; doi:10.3390/diagnostics12112725)
Supplement: Supplementary file 1 [file diagnostics-12-02725-s001.zip › diagnostics-1937500-supplementary.pdf]

Table S1, Parameters of CT scanners of each case

ShorterId	Manufacturer	Model Name	Software Versions	KVP	XRayTube Current	ConvolutionKernel		
						Thin-slice Axial	Thick-slice Axial	Thick-slice Coronal
Training001	TOSHIBA	Aquilion	V3.35ER007	120	190	FC14	FC86	FC14
Training002	TOSHIBA	Aquilion	V3.35ER007	120	180	FC14	FC86	FC14
Training003	Philips	iCT 256	3.2.5.19001	120	414	B	YB	B
Training004	Philips	iCT 256	3.2.5.19001	120	226	B	B	B
Training005	Philips	iCT 256	3.2.5.19001	120	212	B	YB	B
Training006	TOSHIBA	Aquilion	V3.35ER007	120	175	FC14	FC86	FC14
Training007	TOSHIBA	Aquilion	V3.35ER007	120	182	FC14	FC86	FC14
Training008	Philips	Brilliance 64	3.5.5	120	238	B	B	B
Training009	TOSHIBA	Aquilion	V3.35ER007	120	210	FC14	FC14	FC14
Training010	TOSHIBA	Aquilion	V3.35ER007	120	193	FC14	FC14	FC14
Training011	TOSHIBA	Aquilion	V3.35ER007	120	171	FC14	FC14	FC14
Validation001	Philips	Brilliance 64	3.5.5	120	171	A	YB	A
Validation002	Philips	Brilliance 64	3.5.5	120	157	B	B	B
Testing001	SIEMENS	SOMATOM Definition Flash	syngo CT VA48A	100	1236	I50f\2	I70f\2	I31f\2
Testing002	SIEMENS	SOMATOM Definition Flash	syngo CT VA48A	120	986	I50f\2	I70f\2	I31f\2
Testing003	Philips	iCT 256	3.2.5.19001	120	262	B	YB	B
Testing004	SIEMENS	SOMATOM Definition Flash	syngo CT VA48A	120	559	I31f\2	I70f\2	I31f\2
Testing005	SIEMENS	SOMATOM Definition Flash	syngo CT VA48A	120	1168	I50f\2	I31f\2	I31f\2

Testing006	SIEMENS	Sensation Cardiac 64	syngo CT 2006A	120	56	B50f	B60f	B60f
Testing007	SIEMENS	SOMATOM Definition Flash	syngo CT VA48A	120	164	B31f	B30f	B31f
Testing008	SIEMENS	SOMATOM Definition Flash	syngo CT VA48A	120	164	B31f	B30f	B31f
Testing009	SIEMENS	SOMATOM Definition Flash	syngo CT VA48A	120	164	B31f	B30f	B31f
Testing010	SIEMENS	SOMATOM Definition Flash	syngo CT VA48A	120	164	B31f	B30f	B31f
Testing011	SIEMENS	SOMATOM Definition Flash	syngo CT VA48A	120	164	B31f	B30f	B31f
Testing012	SIEMENS	SOMATOM Definition Flash	syngo CT VA48A	120	164	B31f	B30f	B31f
Testing013	SIEMENS	SOMATOM Definition Flash	syngo CT VA48A	120	164	B31f	B30f	B31f
Testing014	SIEMENS	SOMATOM Definition Flash	syngo CT VA48A	120	164	B31f	B30f	B31f
Testing015	Philips	iCT 256	3.2.5.19001	120	60	B	B	B
Testing016	SIEMENS	SOMATOM Definition Flash	syngo CT VA48A	120	164	B31f	B30f	B31f
Testing017	SIEMENS	SOMATOM Definition Flash	syngo CT VA48A	120	164	B31f	B30f	B31f
Testing018	SIEMENS	Sensation Cardiac 64	syngo CT 2006A	120	56	B50f	B60f	B60f
Testing019	SIEMENS	SOMATOM Definition Flash	syngo CT VA48A	120	164	B31f	B30f	B31f
Testing020	SIEMENS	Sensation Cardiac 64	syngo CT 2006A	120	84	B50f	B60f	B60f
Testing021	SIEMENS	SOMATOM Definition Flash	syngo CT VA48A	120	164	B31f	B30f	B31f
Testing022	SIEMENS	SOMATOM Definition Flash	syngo CT VA48A	120	164	B31f	B30f	B31f
